# Menstrual Bleeding Changes Are NORMAL: Proposed Counseling Tool to Address Common Reasons for Non-Use and Discontinuation of Contraception

**DOI:** 10.9745/GHSP-D-18-00093

**Published:** 2018-10-03

**Authors:** Kate H. Rademacher, Jill Sergison, Laura Glish, Lauren Y. Maldonado, Amelia Mackenzie, Geeta Nanda, Irina Yacobson

**Affiliations:** a FHI 360, Durham, NC, USA.; b Population Services International, Washington, DC, USA.

## Abstract

A new family planning counseling tool uses the simple mnemonic device “NORMAL” to help family planning counselors and providers communicate to their clients key messages about menstrual bleeding changes associated with use of hormonal contraception and the copper IUD.

## BACKGROUND

In 2017, an estimated 214 million women of reproductive age living in low-resource settings wanted to avoid pregnancy but were not using a modern method of contraception.^1^ Data from Demographic and Health Surveys conducted between 2005 and 2014 reveal that almost one-third of women cite concerns about side effects or fear of health risks as a reason for non-use of modern contraception.^2^ In addition, nearly 40% of women who want to avoid pregnancy report they used a contraceptive in the past but discontinued use because of method-related issues.^3^ Evidence shows that menstrual bleeding changes associated with contraceptive use contribute to both discontinuation rates and non-use of contraception.^4^^–^^10^ Many women fear that menstrual changes—such as heavier bleeding, prolonged bleeding, irregular bleeding, spotting, and absence of bleeding (amenorrhea)—can lead to negative health consequences, including infertility. In addition, women often perceive that menstruation is a natural sign of femininity; they worry absence of bleeding is a sign of pregnancy; and they fear a build-up of “dirty” or “bad” blood in their bodies. Unsurprisingly, changes in menstrual bleeding are known to impact women's daily lives and relationships with their partners.^10^^–^^18^

Helping women understand the typical bleeding changes associated with the use of modern contraceptive methods could lead to greater acceptance of these changes, increased method uptake, improved satisfaction, and higher continuation rates.^10^^,^^19^ In particular, both health care providers and contraceptive users should understand that changes to menstrual bleeding—including absence of bleeding—due to the use of contraceptive methods will not negatively impact women's health.^20^^,^^21^ A clearer understanding of potential bleeding changes associated with a given contraceptive method and anticipated lifestyle implications may also help women make well-informed decisions about the specific method that best meets their needs.^22^

Understanding the potential bleeding changes associated with various contraceptive methods and potential lifestyle implications could help women make well-informed decisions about the specific method that best meets their needs.

In addition, amenorrhea and oligomenorrhea (infrequent bleeding) associated with certain hormonal contraceptive methods can have important noncontraceptive health benefits as well as lifestyle advantages for some women.^20^^,^^21^ Positioning noncontraceptive attributes as having potential advantages for women, rather than characterizing all menstrual bleeding changes as unpleasant side effects, could potentially lead to increased demand for and satisfaction with hormonal contraceptive methods.^23^

Despite the potential advantages of providing high-quality counseling on these topics to family planning clients, it is unclear the extent to which this happens in the field and what impact these types of messages might have on method uptake or continued use. A recent review by Polis et al.^10^ found few studies that have evaluated whether counseling clients on changes to menses influences method choice or improves continuation rates. The authors noted that development of a counseling tool could help health care providers better communicate with clients about potential bleeding changes associated with contraceptive use. The work described here was undertaken to address this gap.

## CURRENT LANDSCAPE: EXISTING MESSAGES IN INTERNATIONAL TRAINING AND COUNSELING MATERIALS

As a first step, to better understand what current guidance is available to health care providers on how to counsel women about menstrual bleeding changes and contraceptive use, we reviewed counseling, training, and reference materials developed and commonly used by international family planning programs (Table).^24^^–^^34^ Two clinicians independently reviewed each resource to evaluate if and how menstrual bleeding changes were addressed. They examined 6 parameters to determine whether providers were instructed to (1) describe bleeding changes clients should expect with specific contraceptive methods; (2) compare typical bleeding changes among different contraceptive methods; (3) reassure women about menstrual bleeding changes, either general or detailed reassurance; (4) describe strategies to manage inconvenient menstrual changes; (5) provide basic information about menstruation and/or menstrual hygiene; and (6) explain the potential health benefits of oligomenorrhea or amenorrhea. In addition, reviewers also noted whether providers were instructed to describe typical menstrual changes before or after a client selected a specific contraceptive method, and whether tailored information was included for special populations, such as youth or postpartum women. The information was evaluated for 4 contraceptive methods—implants, injectables, the copper intrauterine device (IUD), and the levonorgestrel intrauterine system (LNG IUS)—because of the high likelihood of these methods to change menstrual bleeding patterns.

**TABLE.** Content Related to Menstrual Bleeding Changes in Key International Family Planning Counseling and Training Resources

Resource/Lead Organization(s)	Contraceptive Method	Messages/Content Evaluated							
		Type of Expected Bleeding Changes	Comparison of Bleeding Changes Among Different Methods	When to Address Bleeding Changes	Type of Reassurance Provided^a^	Strategies to Manage Bleeding Changes and Associated Symptoms	Overview of Function and Biological Process of Menstruation	Potential Benefits of Oligomenorrhea or Amenorrhea	Expected Bleeding Changes for Special Populations^b^
*The Balanced Counseling Strategy Plus: A Toolkit for Family Planning Service Providers Working in High HIV/STI Prevalence Settings*^24^/Population Council	Implants	Y	Y	After method initiation	2	N	N	Y	N
	DMPA injectable	Y	N	After method initiation	1	N	N	Y	N
	Copper IUD	Y	N	After method initiation	2	N	N	N/A	N
	LNG IUS	Y	N	After method initiation	2	N	N	Y	N
*Training Resource Package for Family Planning*^25^^c^/ USAID, WHO, and UNFPA	Implants	Y	N	Before method initiation	2	N	N	Y	N
	DMPA injectable	Y	N	Before method initiation	3	Y	N	Y	N
	Copper IUD	Y	N	Before method initiation	2	Y	N	N/A	Y
*Providing Long-Acting Reversible Contraception (LARC) Learning Resource Package (Modular/Facility-Based)*^26^^d^/MCSP, Jhpiego	Implants	Y	N	Before method initiation	2	Y	N	Y	Y
	Copper IUD	Y	N	Before method initiation	2	Y	N	N/A	N
	LNG IUS	Y	N	Before method initiation	2	Y	N	Y	Y
Pathfinder Family Planning Resources^27^^–^^32^^c^/Pathfinder	Implants	Y	N	Before method initiation	3	Y	N	Y	N
	DMPA injectable	Y	N	Before method initiation	2	Y	N	Y	Y
	Copper IUD	Y	N	Before method initiation	2	Y	N	N/A	Y
*Family Planning: A Global Handbook for Providers, 3rd ed.*^33^/WHO and K4Health Project, Johns Hopkins CCP	Implants	Y	N	Before and after method initiation	3	Y	N	Y	N
	DMPA injectable	Y	N	Before and after method initiation	3	Y	N	Y	N
	Copper IUD	Y	N	Before and after method initiation	2	Y	N	N/A	N
	LNG IUS	Y	N	Before and after method initiation	2	N	N	Y	N
*LNG IUS Training Manual for Family Planning*^34^^e^/ICA Foundation	LNG IUS	Y	Y	Before method initiation	2	Y	N	Y	N

Abbreviations: CCP, Center for Communication Programs; DMPA, depot medroxyprogesterone acetate; ICA, International Contraceptive Access; IUD, intrauterine device; LNG IUS, levonorgestrel intrauterine system; K4Health, Knowledge for Health; MCSP, Maternal and Child Survival Program; UNFPA, United Nations Population Fund; USAID, United States Agency for International Development; WHO, World Health Organization.

a 1=none; 2=general "no harm" message; 3=detailed "no harm" message.

b Special populations include youth and postpartum women.

c Method-specific information on the LNG IUS is not included in this resource.

d Method-specific information on DMPA is not included in this resource.

e Information about the LNG IUS is not included in all resources because it is not widely available in developing countries. The ICA Foundation donates free LNG IUS units and has LNG IUS training resources for providers on their website. The ICA Foundation materials were reviewed for information on the LNG IUS specifically and did not include information on implants, DMPA, or the copper IUD.

A key finding from the assessment was that menstrual bleeding changes are insufficiently addressed in the resources reviewed. In general, common bleeding changes, such as heavier or decreased bleeding, and the potential benefits of reduced or no bleeding are addressed in all of the resources evaluated; however, these topics either do not receive much emphasis or little detail is provided. Although resources often instruct providers to reassure women that bleeding changes are not a sign of illness, global evidence demonstrates that women's concerns about bleeding changes are more varied and nuanced.^10^ As such, family planning counselors need to be able to provide clients with general information about bleeding changes as well as communicate information tailored to clients' individual concerns. Results from the assessment of training, counseling, and reference materials are summarized in the Table.

## PROPOSED TOOL

To address gaps in existing guidance and training materials, a multidisciplinary project team from FHI 360 and Population Services International (PSI) developed a simple set of counseling messages about menstrual bleeding changes associated with contraceptive use. The objective of this project, which was funded by the United States Agency for International Development (USAID), was to develop a resource that health care providers could easily incorporate into counseling sessions without substantially increasing time or effort requirements. The primary goals of the tool are to prompt providers to (1) educate women on bleeding changes associated with use of contraception, (2) address common misconceptions and fears about menstrual changes, and (3) increase women's awareness of the potential advantages of reduced menstrual bleeding and/or amenorrhea.

The tool uses the simple mnemonic device “NORMAL”—Normal, Opportunities, Return, Methods, Absence of Menses, and Limit—to help practitioners remember brief messages about menstrual bleeding changes associated with hormonal contraception and the copper IUD and address typical concerns and questions women often have. The NORMAL tool prompts providers to address the following 6 points:

1. **NORMAL – Changes to your menses are NORMAL when you use a contraceptive method.** With this point, providers are encouraged to describe the different types of bleeding changes that women can expect—specifically, changes in volume, duration, and predictability of menses—with use of hormonal contraception and the copper IUD. Providers are also instructed to tell clients that menstrual changes can vary over time with continued use of hormonal contraception.^33^
2. **OPPORTUNITIES – Lighter or no menses can provide OPPORTUNITIES that may benefit your health and personal life.** Providers are prompted to inform clients of the potential health benefits and lifestyle advantages associated with reduced bleeding or amenorrhea. For example, all hormonal contraceptives offer some protection from iron-deficiency anemia, and some methods—such as oral contraceptive pills and the LNG IUS—are used as effective treatments for heavy menstrual bleeding (menorrhagia).^33^ Also, the absence of bleeding or infrequent bleeding can be convenient for women by increasing their ability to participate in educational or work activities, lowering financial costs and the burden of menstrual hygiene management, and reducing disruption of sexual activity.^21^^,^^35^
3. **RETURN – Once you stop using a method, your menses will RETURN to your usual pattern, and your chances of getting pregnant will RETURN to normal.** Providers are encouraged to reassure women that menstrual bleeding changes are not permanent and will not harm their future fertility. For most contraceptive methods, fertility will return rapidly after use of contraception is discontinued. In the case of injectable contraception, return to fertility will likely be delayed for several months after stopping use.^33^
4. **METHODS – Different contraceptive METHODS can lead to different bleeding changes.** Providers should ask a woman about her preferences regarding bleeding changes when she is selecting a method. Bleeding profiles differ across methods, and women's preferences should inform a tailored counseling approach and be incorporated into deciding which method to select.^22^
5. **ABSENCE OF MENSES – If you are using a hormonal method, ABSENCE OF MENSES does not mean that you are pregnant.** Providers should reassure users of hormonal contraception that they should not assume absence of menstrual bleeding is, by itself, a sign of pregnancy. If a woman has other signs of pregnancy while using a hormonal method, or if she misses her menses while using copper IUD, she should talk with her provider or take a pregnancy test.^33^
6. **LIMIT – If changes to your menses LIMIT your daily activities, there are simple treatments available.** If a woman perceives bleeding changes as unpleasant or worrisome, she should be encouraged to talk with her provider about options before she decides to discontinue a method. For example, irregular or heavy bleeding may interfere with women's daily lives or increase their menstrual hygiene management burden.^10^ Simple treatment options are available that can help alleviate troubling physical symptoms.^33^^,^^36^ Additional education and reassurance can also be helpful.^37^

A new counseling tool uses the simple mnemonic device “NORMAL” to help providers remember important messages about menstrual bleeding changes associated with contraception.

Evidence shows that use of acronyms and mnemonic devices can improve clinical practice in a range of fields, and that the introduction of simple evidence-based checklists and job aids for family planning providers can lead to increased contraceptive use in low-resource settings.^38^^–^^41^ Two mnemonic devices have been commonly used in international family planning programs to train providers on appropriate sequencing of counseling steps: GATHER, which stands for Greet, Ask, Tell, Help, Explain, and Return, and REDI, which stands for Rapport building, Exploration, Decision making, and Implementing the decision.^42^^,^^43^ The NORMAL tool complements these frameworks as well as other commonly used counseling approaches.

Research has demonstrated that when delivering information about a health intervention, there is a risk of inadvertently reinforcing misconceptions among the target population.^44^^,^^45^ As such, all messages included in the NORMAL tool are framed in positive language that avoids repeating inaccurate information. Although only a subset of contraceptive methods was included in the initial review of international counseling and training resources described earlier, the NORMAL tool was designed to be relevant and applicable for all hormonal methods and the copper IUD. Providers who use the tool are instructed to use it both before and after a client selects a contraceptive method; they are also prompted to remind their clients to return for additional counseling and/or treatment if they have concerns about bleeding changes after method initiation.

In 2017, the team that developed the NORMAL tool solicited feedback on a preliminary draft from private- and public-sector health care providers in Haiti and Zambia during family planning training workshops, and from PSI and FHI 360 reproductive health staff in Nigeria and Zambia during project meetings. The format and content of the tool were then revised based on the input received. A final version of the NORMAL tool (Figure) is also available in French, Portuguese, and Spanish; the term “NORMAL” is used in all 3 translated versions, with minor adjustments to ensure the translated content is accurate and appropriate. (To download the English and translated versions, see www.fhi360.org/resource/normal-counseling-tool-menstrual-bleeding-changes-job-aid.) Because the tool is meant to prompt providers to address key points and is not intended to be a script that is read verbatim, the expectation is that providers will be able to address the same topics using local languages with clients.

**FIGURE:**
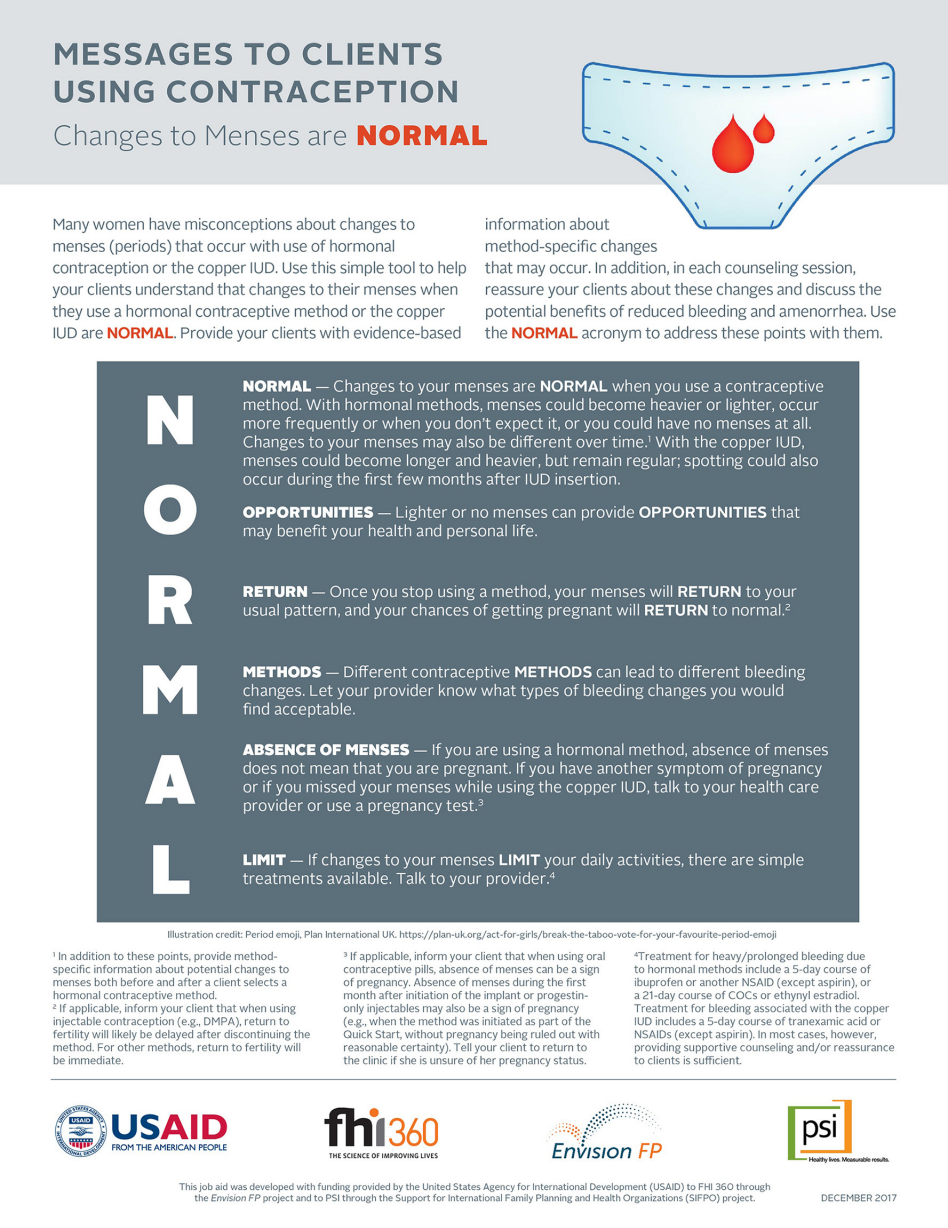
NORMAL Counseling Tool Abbreviations: COCs, combined oral contraceptives; DMPA, depot medroxyprogesterone acetate; IUD, intrauterine device; NSAID, nonsteroidal anti-inflammatory drug.

## NEXT STEPS

Changing women's knowledge and attitudes about menstrual bleeding changes associated with contraceptive use may enhance acceptability of modern contraceptive methods, reduce discontinuation rates among current users, and increase use among women with unmet need for contraception. The NORMAL counseling tool is designed to reduce common myths and misconceptions among women, improve women's knowledge of bleeding changes, and increase women's interest in the noncontraceptive benefits associated with oligomenorrhea or amenorrhea. This tool could be incorporated into facility- or community-based provision of family planning, and could be included in preservice and on-the-job training for providers.

The NORMAL tool may help enhance acceptability of modern contraceptive methods, reduce discontinuation rates among current users, and increase use among women with unmet need for contraception.

Before the tool is implemented on a wide scale, additional research is needed to further evaluate the feasibility and effectiveness of incorporating the NORMAL tool into family planning counseling sessions, women's comprehension of these messages, and the ultimate impact on changing providers' and women's attitudes and behaviors. In 2018, the NORMAL tool will be evaluated as part of a USAID-funded study in Malawi. Additionally, development of the tool was based on the initial review of counseling and training tools developed by international groups; an important next step would be to review national family planning guidelines and training curricula to determine if and how menstrual bleeding changes are addressed in those documents. Following that review, national stakeholders could be encouraged to incorporate the NORMAL tool into these resources, pending positive evaluation results.

## Notes

**Peer Reviewed**

**Funding**: This work was made possible by the generous support of the American people through the U.S. Agency for International Development (USAID).

## References

[1] Guttmacher Institute. Adding It Up: Investing in Contraception and Maternal and Newborn Health, 2017. [Fact Sheet.] New York: Guttmacher Institute; 2017. https://www.guttmacher.org/sites/default/files/factsheet/adding-it-up-contraception-mnh-2017.pdf. Accessed August 8, 2018.

[2] Sedgh G Ashford LS Hussain R. Unmet Need for Contraception in Developing Countries: Examining Women's Reasons for Not Using a Method. New York: Guttmacher Institute; 2016. https://www.guttmacher.org/report/unmet-need-for-contraception-in-developing-countries. Accessed August 8, 2018.

[3] Castle S Askew I. Contraceptive Discontinuation: Reasons, Challenges, and Solutions. New York: Population Council; 2015. http://ec2-54-210-230-186.compute-1.amazonaws.com/wp-content/uploads/2016/02/FP2020_ContraceptiveDiscontinuation_SinglePage_Revise_02.15.16.pdf. Accessed August 8, 2018.

[4] Belsey E Farley TM. The analysis of menstrual bleeding patterns: A review. Contraception. 1988;38(2):129–156. 10.1016/0010-7824(88)90035-2. 3048871

[5] Berlan E Mizraji K Bonny AE. Twelve-month discontinuation of etonogestrel implant in an outpatient pediatric setting. Contraception. 2016;94(1):81–86. 10.1016/j.contraception.2016.02.030. 26948183

[6] Diedrich JT Desai S Zhao Q Secura G Madden T Peipert JF. Association of short-term bleeding and cramping patterns with long-acting reversible contraceptive method satisfaction. Am J Obstet Gynecol. 2015;212(1):50.e1–50.e8. 10.1016/j.ajog.2014.07.025. 25046805 PMC4275360

[7] Glasier A. Implantable contraceptives for women: effectiveness, discontinuation rates, return of fertility, and outcome of pregnancies. Contraception. 2002;65(1):29–37. 10.1016/S0010-7824(01)00284-0. 11861053

[8] Salem RM Setty V Williamson RT Schwandt H. When contraceptives change monthly bleeding. Popul Rep J. 2006;54(1):3–19. 17361533

[9] Tolley E Loza S Kafafi L Cummings S. The impact of menstrual side effects on contraceptive discontinuation: findings from a longitudinal study in Cairo, Egypt. Int Fam Plan Perspect. 2005;31(01):15–23. 10.1363/3101505. 15888405

[10] Polis CB Hussain R Berry A. There might be blood: a scoping review on women's responses to contraceptive-induced menstrual bleeding changes. Reprod Health. 2018;15(1):114. 10.1186/s12978-018-0561-0. 29940996 PMC6020216

[11] Das M. Socio-cultural aspects of menstruation: an anthropological purview. East Anthropol. 2008;61(2):227–240.

[12] Edelman A Lew R Cwiak C Nichols M Jensen J. Acceptability of contraceptive-induced amenorrhea in a racially diverse group of US women. Contraception. 2007;75(6):450–453. 10.1016/j.contraception.2007.02.005. 17519151

[13] Estanislau do Amaral MC Hardy E Hebling EM Faúndes A. Menstruation and amenorrhea: opinion of Brazilian women. Contraception. 2005;72(2):157–161. 10.1016/j.contraception.2005.02.013. 16022856

[14] Glasier AF Smith KB van der Spuy ZM. Amenorrhea associated with contraception—an international study on acceptability. Contraception. 2003;67(1):1–8. 10.1016/S0010-7824(02)00474-2. 12521650

[15] Newton V Hoggart L. Menstruation and contraception: social and cultural issues on young women's decision making. Contraception. 2015;92(4):403. 10.1016/j.contraception.2015.06.193

[16] Snow R Hardy E Kneuper E Hebling EM Hall G. Women's responses to menses and nonbleeding intervals in the USA, Brazil and Germany. Contraception. 2007;76(1):23–29. 10.1016/j.contraception.2007.03.008. 17586132

[17] World Health Organization Task Force on Psychosocial Research in Family Planning, Special Programme of Research, Development and Research, Training in Human Reproduction. A cross-cultural study of menstruation: implications for contraceptive development and use. Stud Fam Plann. 1981;12(1):3–16. 10.2307/1965859. 7466889

[18] Castle S. Factors influencing young Malians' reluctance to use hormonal contraceptives. Stud Fam Plann. 2003;34(3):186–199. 10.1111/j.1728-4465.2003.00186.x. 14558321

[19] Halpern V Grimes DA Lopez L Gallo MF. Strategies to improve adherence and acceptability of hormonal methods for contraception. Cochrane Database Syst Rev. 2006;2006(1):CD004317. 16437483 10.1002/14651858.CD004317.pub2

[20] Association of Reproductive Health Professionals (ARHP). Health Matters: Understanding Menstrual Suppression. Washington, DC: ARHP; 2008 https://www.arhp.org/uploadDocs/understandingmenstrualsuppression.pdf. Accessed August 8, 2018.

[21] Lin K Barnhart K. The clinical rationale for menses-free contraception. J Womens Health (Larchmt). 2007;16(8):1171–1180. 10.1089/jwh.2007.0332. 17937570

[22] Rominski SD Morhe ESK Maya E Manu A Dalton VK. Comparing women's contraceptive preferences with their choices in 5 urban family planning clinics in Ghana. Glob Health Sci Pract. 2017;5(1):65–74. 10.9745/GHSP-D-16-00281. 28179370 PMC5493451

[23] Rademacher KH Solomon M Brett T. Expanding access to a new, more affordable levonorgestrel intrauterine system in Kenya: service delivery costs compared with other contraceptive methods and perspectives of key opinion leaders. Glob Health Sci Pract. 2016;4(suppl 2):S83–S93. 10.9745/GHSP-D-15-00327. 27540128 PMC4990165

[24] Population Council. The Balanced Counseling Strategy Plus: A Toolkit for Family Planning Service Providers Working in High HIV/STI Prevalence Settings. 3rd ed. New York: Population Council; 2016. http://www.popcouncil.org/research/the-balanced-counseling-strategy-plus-a-toolkit-for-family-planning-service. Accessed August 9, 2018.

[25] United States Agency for International Development, World Health Organization, United National Population Fund. Training resource package for family planning. TRP Website. https://www.fptraining.org/. Accessed August, 2017.

[26] Maternal and Child Survival Program (MCSP). Providing Long-Acting Reversible Contraception (LARC) Learning Resource Package (Modular/Facility-Based). Washington, DC: MCSP; 2017. http://resources.jhpiego.org/resources/Modular_LARC_LRP. Accessed August 8, 2018.

[27] Pathfinder International. Cue Cards for Counseling Adolescents on Contraception. Watertown, MA: Pathfinder International, 2016. https://www.pathfinder.org/wp-content/uploads/2016/12/Adolescent-Contraception-Cue-Cards.pdf. Accessed August 17, 2018.

[28] Pathfinder International. Contraceptive Implants Clinical Training: Trainers Guide. Watertown, MA: Pathfinder International, 2016. https://www.pathfinder.org/publications/implants-training/. Accessed August 17, 2018.

[29] Pathfinder International. Contraceptive Implants Clinical Training: Participant Handouts. Watertown, MA: Pathfinder International, 2016. https://www.pathfinder.org/publications/implants-training/. Accessed August 17, 2018.

[30] Pathfinder International. Comprehensive Reproductive Health and Family Planning Training Curriculum, Module 6: DMPA Injectable Contraceptive. Watertown MA: Pathfinder International; 1997. https://www.pathfinder.org/wp-content/uploads/2016/10/Module-6-DMPA-Injectables.pdf. Accessed August 17, 2018.

[31] Pathfinder International. Intrauterine Devices: Trainer's Guide. 2nd ed. Watertown MA: Pathfinder International; 2008. https://www.pathfinder.org/wp-content/uploads/2016/11/Intrauterine-Devices-Trainers-Guide-Second-Edition.pdf. Accessed August 17, 2018.

[32] Pathfinder International. Intrauterine Devices: Participant's Guide. 2nd ed. Watertown MA: Pathfinder International; 2008. https://www.pathfinder.org/wp-content/uploads/2016/11/Intrauterine-Devices-Participants-Guide-Second-Edition.pdf. Accessed August 17, 2018.

[33] World Health Organization (WHO); Johns Hopkins Center for Communication Programs (CCP), Knowledge for Health Project. Family Planning: A Global Handbook for Providers. 3rd ed. Baltimore and Geneva: CCP and WHO; 2018. http://apps.who.int/iris/handle/10665/260156. Accessed August 8, 2018.

[34] International Contraception Access (ICA) Foundation. LNG IUS Training Manual for Family Planning. Turku, Finland; 2004. http://www.ica-foundation.org/ICA%20_LNG_manual.pdf.

[35] Hardy E Hebling EM de Sousa MH Kneuper E Snow R. Association between characteristics of current menses and preference for induced amenorrhea. Contraception. 2009;80(3):266–269. 10.1016/j.contraception.2009.06.010. 19698819

[36] Mansour D Bahamondes L Critchley H Darney P Fraser IS. The management of unacceptable bleeding patterns in etonogestrel-releasing contraceptive implant users. Contraception. 2011;83(3):202–210. 10.1016/j.contraception.2010.08.001. 21310280

[37] Rodriguez J Abutouk M Roque K Sridhar A. Personalized contraceptive counseling: helping women make the right choice. Open Access J Contracept. 2016;7:89–96. 29386940 10.2147/OAJC.S81546 PMC5683162

[38] Bellezza FS. Mnemonic devices: classification, characteristics, and criteria. Rev Educ Res. 1981;51(2):247–275. 10.3102/00346543051002247

[39] Qureshi A Rizvi F Syed A Shahid A Manzoor H. The method of loci as a mnemonic device to facilitate learning in endocrinology leads to improvement in student performance as measured by assessments. Adv Physiol Educ. 2014;38(2):140–144. 10.1152/advan.00092.2013. 25039085 PMC4056179

[40] Stanback J Diabate F Dieng T Duarte de Morales T Cummings S Traoré M. Ruling out pregnancy among family planning clients: the impact of a checklist in three countries. Stud Fam Plann. 2005;36(4):311–315. 10.1111/j.1728-4465.2005.00073.x. 16395948

[41] Stanback J Qureshi Z Sekadde-Kigondu C Gonzalez B Nutley T. Checklist for ruling out pregnancy among family-planning clients in primary care. Lancet. 1999;354(9178):566. 10.1016/S0140-6736(99)01578-0. 10470704

[42] Rinehart W Rudy S Drennan M. GATHER guide to counseling. Popul Rep J. 1998;26(4):1–31. www.k4health.org/sites/default/files/j48.pdf. Accessed September 14, 2018. 10096107

[43] The Access, Quality, and Use in Reproductive Health (ACQUIRE) Project. Counseling for Effective Use of Family Planning. Participant Handbook. Washington, DC: EngenderHealth; 2008. https://www.engenderhealth.org/files/pubs/acquire-digital-archive/10.0_training_curricula_and_materials/10.2_resources/fp_curric_ph_main_text.pdf. Accessed August 9, 2018.

[44] Lewandowsky S Ecker UK Seifert CM Schwarz N Cook J. Misinformation and its correction: continued influence and successful debiasing. Psychol Sci Public Interest. 2012;13(3):106–131. 10.1177/1529100612451018. 26173286

[45] PATH. Countering myths and misperceptions about contraception. Outlook on Reproductive Health. Seattle. Seattle, WA: PATH; 2015. https://path.azureedge.net/media/documents/RH_outlook_myths_mis_june_2015.pdf. Accessed August 17, 2018.

